# Effect of motivational interviewing on the weight-affecting lifestyle and body mass index in overweight women with polycystic ovary syndrome: a randomized clinical trial

**DOI:** 10.61622/rbgo/2026rbgo26

**Published:** 2026-08-20

**Authors:** Fereshteh Khaleghi Nejad, Behnaz Enjezab, Reyhane Sefidkar, Fatemeh ZareMobini

**Affiliations:** 1 Shahid Sadoughi University of Medical Sciences School of Nursing and Midwifery Department of Midwifery Yazd Iran Department of Midwifery, School of Nursing and Midwifery, Shahid Sadoughi University of Medical Sciences, Yazd, Iran.; 2 Shahid Sadoughi University of Medical Sciences Comprehensive Research Institute for Maternal and Child Health Research Center for Nursing and Midwifery Care Yazd Iran Research Center for Nursing and Midwifery Care, Comprehensive Research Institute for Maternal and Child Health, Shahid Sadoughi University of Medical Sciences, Yazd, Iran.; 3 Shahid Sadoughi University of Medical Sciences School of public health Department of Biostatistics and Epidemiology Yazd Iran Center for Healthcare Data Modeling, Department of Biostatistics and Epidemiology, School of public health, Shahid Sadoughi University of Medical Sciences, Yazd, Iran.; 4 Shahid Sadoughi University of Medical Sciences School of Nursing and Midwifery Department of Midwifery Yazd Iran Research Center for Nursing and Midwifery Care, Comprehensive Research Institute for Maternal and Child Health, Department of Midwifery, School of Nursing and Midwifery, Shahid Sadoughi University of Medical Sciences, Yazd, Iran.

**Keywords:** Body mass index, Lifestyle, Motivational interviewing, Polycystic ovary syndrome, Overweight

## Abstract

**Objective:**

The study investigates the effect of Motivational Interviewing (MI) on the lifestyle affecting weight and body mass index in overweight women with Polycystic Ovary Syndrome (PCOS).

**Methods:**

This is a parallel clinical trial, conducted in 2024 in Landeh, Iran; with sixty overweight women with PCOS were randomly assigned to intervention and control groups. In the intervention group, MI counselling was implemented for five 90-minute sessions. The control group was provided with an educational booklet on PCOS. Participants’ weight and Body Mass Index (BMI) were measured and calculated at the beginning of the study and twelfth week after the intervention. Also, data were collected using demographic and Weight-affecting Lifestyle Questionnaire (WELSQ) at baseline and twelfth week follow-up. Data were analyzed using SPSS version 26 (P< 0.05).

**Results:**

In the intervention Group, the mean weight-affecting lifestyle score significantly increased from 121.83±17.44 to 142.03±14.48 at 12 weeks (P<0.001). The results showed that there is a significant difference between the average weight between the two groups after the intervention (p=0.02). In the intervention group the mean BMI score decreased from 27.27±1.16 to 26.50±1.32 at 12 weeks (P<0.001). The results showed a statistically significant difference in the mean score of weight-affecting lifestyle (p=0.003) and BMI (p=0.03) between the two groups after the intervention.

**Conclusion:**

Counseling based on MI improved weight-affecting lifestyle and BMI in women with PCOS. So, it is recommended that this method be employed to promote weight-affecting lifestyle and, as a result, weight control and help better management in women with PCOS.

**Iranian Registry of Clinical Trials**: IRCT20231121060125N1

## Introduction

Polycystic ovary syndrome (PCOS) is the most common endocrine disorder in women, and due to its burden, global attention is needed to control it.^(1,2)^ The prevalence of PCOS worldwide is estimated to be 5-17%.^(3)^ Although there is no single criterion for diagnosing this syndrome, it is diagnosed based on a combination of clinical, laboratory, and ultrasound findings.^(4,5)^ In addition to an increased risk of reproductive problems such as infertility, endometrial and breast cancer, and premature menopause, women with this syndrome are at increased risk of psychological problems such as depression, low self-esteem, anxiety, and a group of metabolic disorders including impaired glucose tolerance, hypertension, and cardiovascular disease.^(6-9)^ According to the results of studies, lifestyle and weight play a significant role in the occurrence of this disease.^(10)^ More than 50% of women with PCOS are overweight.^(4)^

Obesity is one of the most common chronic metabolic diseases worldwide.^(11)^ Systematic reviews and numerous meta-analyses from China, the United Kingdom, and Norway have shown that overweight and obesity increase all-cause mortality by 5% to 9%, respectively, and increase the risk of sudden cardiac death by 1.2 to 1.5 times.^(12,13)^ It also increases the risk of insulin resistance,^(14)^ sensitizes follicular membrane cells to luteinizing hormone (LH) stimulation, and increases ovarian androgen secretion, exacerbating hyperandrogenism. On the other hand, the metabolic activities of these cells, in turn, affect the increase in adipose tissue and exacerbate obesity. This cycle continues to aggravate PCOS; consequently, managing this disease is of particular importance to achieving weight loss.^(15)^ In overweight patients with PCOS, body fat is typically accumulated in an android (apple-shaped or abdominal) pattern; this type of fat distribution is associated with metabolic disorders such as type 2 diabetes, dyslipidemia, metabolic syndrome, as well as hormonal disorders such as hyperandrogenism and anovulation.^(16,17)^

According to the clinical guidelines for PCOS, lifestyle interventions such as dietary changes and physical activity are first-line treatments.^(18)^ The significant correlation between weight loss of even 5 to 10% of body weight and improvements in metabolic and hormonal indices and increased fertility can be attributed to the reduction in abdominal circumference, visceral fat, and insulin resistance.^(19)^ Various studies have shown that in addition to improving fertility and clinical symptoms of PCOS, lifestyle change programs can also be effective in reducing cardiovascular risk factors.^(20,21)^

Although overweight and obesity are major public health problems that contribute to impaired life quality related to physical and mental health and increased morbidity and mortality, more than half of overweight individuals are not active enough and adhere to lifestyle interventions inadequately.^(22,23)^ Some previous studies have cited lack of sufficient motivation, physical barriers such as health problems, weight, and back pain, and psychosocial barriers such as embarrassment, self-blame, lack of immunity, and lack of time as barriers to weight loss in overweight and obese individuals.^(24,25)^

According to research, motivation theory is one of the appropriate predictors of behavior change for lifestyle changes such as diet and physical activity. Motivation theory shows how individuals behave when faced with a health-threatening factor.^(26)^ Motivational Interviewing (MI) is one of the counseling methods for behavior change. It is a client-centered and direct method to increase individuals’ intrinsic motivation to change behavior by examining and resolving ambiguities. MI strengthens intrinsic motivation to change behavior and creates possible solutions by identifying potential barriers to change. It also provides coherent techniques to support behavior change.^(27,28)^

Studies show that the effectiveness of conventional behavior change methods for managing diseases requiring lifestyle modifications has not been satisfactory.^(27)^ For instance, in one study, 50% of participants in a physical activity program discontinued physical activity within 6 months and only 25% successfully achieved their weight loss goals after 18 months.^(28-30)^ A study showed that implementing the motivational interviewing method leads to greater weight loss.^(31)^ The results of another study also showed that MI increased self-efficacy and reduced BMI in overweight women.^(30)^ Despite the positive results, the study by Christie et al. reported that combining MI and standard weight loss training methods did not lead to increased adherence to the program and, ultimately, weight loss.^(32)^ Therefore, considering the importance of adopting a healthy lifestyle and losing weight in women with PCOS, and on the other hand, the need to motivate these women to modify their behavior, as well as the limited number of studies in this field, the present research team designed the aforementioned study to determine the effect of MI on the weight-affecting lifestyle and BMI in overweight women with PCOS.

## Methods

The present study is a parallel clinical trial study conducted as a pre-test and post-test from January 15, 2024, to April 20, 2024. The research population included overweight women with PCOS referred to the Shahid Rezaei Specialized Clinic in Landeh, Iran. The first researcher selected 60 women from among 90 eligible women who had a file in the Shahid Rezaei Specialized Clinic in Landeh, based on the inclusion and exclusion criteria. To conduct the research, informed consent was obtained from all the participants. The required sample size in each group was obtained based on Meybodi's study^(30)^ and considering significance level of 0.05, power of the test of β-1 = 0.80, type II error level of 0.02 and taking into account 20% attrition, was 30 people in each group.

The participants were randomly assigned to experimental (MI) and control groups (educational booklet) using online software (www.Random.org/sequences) and with due regard for concealment. Inclusion criteria were; Women with PCOS based on the Rotterdam criteria, BMI greater than 25 kg/m2, Ages 15-49 years. Exclusion criteria were; Being pregnant or undergoing assisted reproductive treatment, the presence of severe mental disorders (such as psychosis or schizophrenia under drug treatment based on information in the medical record or his/her statements, Limitations on physical activity, Diseases that require a special diet (such as phenylketonuria). Due to the explanation of the objectives, there was no dropout in this study. The study instrument included demographic and weight-affecting lifestyle questionnaires (WELSQ) completed by participants in both groups before and 12 weeks after the intervention. Demographic information questionnaire included questions on age, height, weight, BMI, marital status, level of education, occupation, duration of disease, number of children, and history of infertility. WELSQ scale was developed by Clark et al. to measure self-control ability among obese individuals seeking treatment (Gatica-Saavedra et al.).^(33)^ It includes 20 questions and aims to measure the ability to resist eating in different situations. The responses are ranked on a ten-point Likert scale, measuring five dimensions (negative emotions, availability, social pressure, physical discomfort, and positive activities). Each dimension will have a score ranging from 10 to 40. A higher score indicates greater confidence in resisting the urge to eat in different situations and conditions.^(33)^ In the study by Ahmadipour et al.,^(34)^ content and construct validity were evaluated in order to examine the validity of the instrument. In content validity, four psychology experts’ opinions on the questionnaire were obtained. Regarding construct validity, two methods were used: internal correlation of each factor with the total score of the questionnaire and confirmatory factor analysis. Its construct validity was also confirmed. Moreover, the reliability of the questionnaire was calculated using Cronbach's alpha coefficient. The alpha value for each domain was between 0.71 and 0.78.^(34)^

Using a SECA digital scale model 301 in the clinic, the subjects’ height, weight, and BMI were measured, calculated, and recorded by a calculator in the center. It should be noted that in order to measure weight and prevent measurement errors, all participants were placed on a fixed scale with bare feet. Weight was measured in the early morning, after emptying the bladder, before having breakfast or any drink, and during or one week before menstruation. The scale was placed on a flat surface. Similar clothing was used for both weighing sessions (before and after the intervention). In the intervention group, MI was conducted in 5 sessions of 90 minutes and groups of 10 individuals. The intervention in the experimental group was MI, which was presented in person. The details of the intervention are presented in chart 1. The experimental group was divided into three groups of 10 individuals for training. Classes for each group were held in five sessions on Saturdays, Mondays, and Wednesdays from 10:00 to 12:00 using PowerPoint and lectures. The participants in the experimental group were coordinated with the days and times of the MI sessions by receiving text messages. Both experimental and control groups received standard treatments for women with PCOS available at the center. The control group was provided with an educational booklet about lifestyle.

**Chart 1:** The content of motivational interviewing* approach sessions

Session	Goals	Content	Homework
1	Getting to know the researcher and the research process, filling out the first-stage questionnaire and answering the questions related to the questionnaire, filling out the informed consent form	Introduction and introductory talks, determining the number of sessions and the duration of each session, the frequency of sessions, the rules, and goals of the sessions, completing the pre-test form (demographic information questionnaire, lifestyle questionnaire), determining the list of the individual's goals for participating in the counseling sessions, giving a notebook and pen to each participant.	Notes on group rules and goals
2	Learning about behavior and how to change behavior, thinking about the benefits and harms of changing behavior	Discovering the positive and negative aspects of maintaining and changing behavior, discussing how changes occur in the client's attitude and intention to change their lifestyle and behavior, how to create change in other people, practicing brainstorming the short-term and long-term benefits and drawbacks of changing lifestyle, examining the importance, confidence, priorities, and external or internal motivations for changing lifestyle attitudes.	Summary note from the session 2. After identifying the stages of behavior change, write down a few examples of health-related behaviors and note what stage of behavior change for that behavior you are in.
3	Learning to define values and writing down each person's life values based on priority	Defining values and practicing prioritizing values, identifying conflicts between values and current behavior, and practicing the driving force or engine of change.	Taking notes on the summary of the third session Completing the values worksheet Determining what is most valuable to them Identifying behaviors that harm the person's desired value
4	Learning the pros and cons of motivations and defining failures and setbacks	Practicing balancing decision-making about intrinsic or extrinsic motivations and mentalizing the pros and cons of extrinsic and intrinsic motivations, reframing and defining the client's failures and setbacks from their perspective.	Taking notes on the summary of the fourth session
5	Summarizing the content, resolving ambiguities, and reviewing the goals	Calling on the client's personal capabilities, reviewing the list of goals written in the first session, answering all questions, summarizing and concluding the exercises of the previous sessions in the form of a vision exercise, preparing to start the lifestyle change program, and determining the post-test date.	Taking notes for the fifth session On a scale of 0 to 10, rate your confidence about changing your lifestyle behavior.

Rahimi A, Taheri M, Dafei M, ZareMobini F. The effect of motivational interviewing on attitude and intention for childbearing in healthcare provider women. BMC Psychol. 2025;13(1):303.

Data were statistically analyzed using SPSS (SPSS, Inc., Chicago, IL, USA) software version 26 after being extracted from the questionnaire. Descriptive statistics were used to present and describe the information, prepare tables, and calculate percentages, means, and standard deviations. Based on the Shapiro-Wilk test, the assumption of normal distribution of quantitative variables in intervention and control groups was confirmed (p<0.05). Therefore, the independent t-test and the chi-square test were used to determine the quantitative variables between the two groups and the difference between the qualitative variables between the two groups, respectively. The independent t-test was used to determine the mean difference between the two groups at baseline and 12 weeks after the intervention. Moreover, the paired t-test was used to find the difference in mean scores in each group before and 12 weeks after the intervention. The significance level of the tests was considered less than 0.05.

This study was approved by the Research Ethics Committee of Shahid Sadoughi University of Medical Sciences, Iran (IR.SSU.REC.1402.062). Before the intervention, oral and written consent were obtained from the participant. All participants were informed of the study goal, method, and the voluntary nature of the research and were assured of the confidentiality of their information. Informed and voluntary consent was obtained from participants in both groups.

## Results

According to the results in table 1, there was no significant difference between the control and intervention groups in terms of qualitative and quantitative demographic variables, including demographic information questionnaire included questions on age, height, weight, BMI, marital status, level of education, occupation, duration of disease, number of children, and history of infertility (p >0.05). The mean age of the study participants was 30 years. The mean duration of disease of the women participating in the study was 3 years. Most of the participants were married housemakers with a diploma or higher education.

**Table 1:** Comparison of participants’ demographic characteristics (qualitative and quantitative variables) in the two group

Qualitative variables group		*p-value	Intervention group		Control group	
			Percentage	Number	Percentage	Number
Education level	Below Diploma	p=0.71	20	6	6.7	2
	Diploma		33.3	10	53.3	16
	University		46.7	14	40	12
Marital Status	Single	p=1	13.3	4	13.3	4
	Married		86.7	26	86.7	26
Occupation	Housewife	p=0.39	76.7	23	66.7	20
	Employed		23.3	7	33.3	10
History of infertility	Yes	p=1	80	22	80	22
	No		20	4	20	4
**Quantitative variables**		**p-value	**Mean**± **standard deviation**		**Mean** ± **standard deviation**	
Age (years)		p=0.96	30.3 ± 3.23		30.27 ± 2.98	
Duration of disease (years)		p=0.59	3.53 ± 1.38		3.72 ± 1.28	
Weight		p=0.65	64.7 ± 6.2		66.5 ± 5.9	
Height		p=0.71	164.8 ± 4.5		165.3 ± 4.2	

Independent t-test results showed no statistically significant difference between the mean weight-affecting lifestyle score between the two groups before the intervention (p=0.30). These results also showed a significant difference between the mean weight-affecting lifestyle score between the two groups after the intervention (p=0.003). The paired t-test was also used to compare the mean weight-affecting lifestyle score before and after the intervention in the experimental and control groups. The results showed a significant difference between the mean weight-affecting lifestyle score before and after the intervention in the experimental group (p<0.001); however, the difference was not significant in the control group (p=0.21). The independent t-test was also used to examine the effect of the intervention on changes in the weight-affecting lifestyle score (after the intervention - before the intervention). The results of this test showed that the intervention had a significant effect on changes in the mean weight-affecting lifestyle score (p<0.001) (Table 2).

**Table 2:** Determination and comparison of the mean score weight-affecting lifestyle at baseline and 12 weeks after intervention in the two groups

Variable	Group	Baseline (n=30)	12 weeks after the intervention (n=30)	Average changes (n=30)	p-value*
Weight-affecting lifestyle	Intervention	121.83±17.44	142.03 ± 14.48	20.20±13.66	p<0.001
	Control	136.67±19.58	137.73± 17.54	1.06±4.60	p=0.21
p-value**		p=0.30	p=0.003	p<0.001	

* Paired samples t-test

** Independent Samples t-test

Independent t-test results showed that there is no statistically significant difference between the average weight in the two groups before the intervention (p=0.24). Also, the result of the independent t-test showed that there is a significant difference between the average weight between the two groups after the intervention (p=0.02). Also, a significant difference was observed between the mean average weight score before and after the intervention in the experimental group (p<0.001) (Table 3).

**Table 3:** Determination and comparison of the mean score of weight at baseline and 12 weeks after intervention in the two groups

Variable	Group	Baseline (n=30)	12 weeks after the intervention (n=30)	Average changes (n=30)	p-value*
Weight	Intervention Control	64.6±7.20	61.97 ± 6.05	-2.0±2.73	p<0.001
		66.5±5.99	67.6± 8.20	0.51±0.25	p=0.09
p-value**		p=0.24	p=0.02	p<0.001	

* Paired samples t-test

** Independent Samples t-test

Independent t-test results showed no significant difference between the mean BMI between the two groups before the intervention (p=0.80). These results also showed a significant difference between the mean BMI score between the two groups after the intervention (p=0.03). Also, a significant difference was observed between the mean BMI score before and after the intervention in the experimental group (p<0.001); however, the difference was not significant in the control group (p=0.16). The independent t-test was also used to examine the effect of the intervention on changes in BMI (after the intervention - before the intervention). The results of this test showed that the intervention had a significant effect on changes in the mean BMI (p<0.001) (Table 4).

**Table 4:** Determination and comparison of the mean score of BMI at baseline and 12 weeks after intervention in the two groups

Variable	Group	Baseline (n=30)	12 weeks after the intervention (n=30)	Average changes (n=30)	p-value*
BMI	Intervention Control	27.27±1.16	26.50 ± 1.32	-0.0±76.58	p<0.001
		27.19±1.33	27.20± 2.36	0.0±8.33	p=0.16
p-value**		p=0.80	p=0.03	p<0.001	

* Paired samples t-test

** Independent Samples t-test

## Discussion

The aim of this study was to investigate the effect of MI on the weight-affecting lifestyle and BMI in overweight and obese women with PCOS. The results indicated that MI had a significant effect on improving the weight-affecting lifestyle and reducing weight and BMI in overweight women with PCOS. Given that within the scope of the research team's investigation, similar and inconsistent results have been obtained with the results of the present study, the present study results were discussed with consistent and inconsistent studies.

MI is more effective than conventional training in increasing self-efficacy for eating behavior as a predictor of the success of weight loss programs.^(30)^ Regarding the weight-related lifestyle self-efficacy subscales, group MI is more effective than conventional training in strengthening and increasing self-efficacy when experiencing negative emotions, access to food, social pressures, physical discomfort, and engaging in positive and fun activities.^(35)^ One reason for the limited effectiveness of conventional weight loss programs is the presence of multiple psychological and environmental barriers to behavior change. These include stress, depression, negative emotions, lack of motivation or its maintenance, and limited access to healthy food options or supportive social environments. Such barriers often undermine individuals’ self-efficacy and adherence to lifestyle interventions. Strengthening self-efficacy and addressing these challenges through tailored behavioral strategies like motivational interviewing can significantly improve outcomes. In a randomized controlled trial, Hardcastle et al. ^(36)^ demonstrated that MI was more effective than standard nutrition education in promoting sustained weight loss, physical activity, and cardiovascular health over a 12-month follow-up period.^(36)^

In this regard, Resnicow et al.^(37)^ reviewed conceptual and empirical evidence supporting the use of motivational interviewing in pediatric populations with obesity. They emphasized that MI can be effectively adapted to enhance engagement, reduce BMI, and improve psychological outcomes such as self-esteem in children and adolescents. The authors suggested that MI's client-centered approach may be particularly beneficial in addressing ambivalence and promoting sustainable behavior change in younger age groups.^(37)^

Moeller et al.^(38)^ conducted a randomized trial study to determine the effect of MI on obese women with polycystic ovary syndrome. The results of the study showed that no significant difference was observed between the intervention and control groups immediately after the intervention and at the follow-up time after 6 months in terms of weight, BMI, and quality of life.^(38)^ The results of the present study were not consistent with Moeller et al.'s study.^(38)^ In the present study, overweight women with a BMI above 25 were included, and the mean BMI of the participants in the study was approximately 27. In Moeller's study,^(38)^ women with a BMI above 30 were included. Interventions through MI in overweight and non-obese women may have positive effects on BMI. In another study by Mahoney,^(39)^ conducted to determine the effect of lifestyle modification intervention in overweight and obese infertile women with PCOS, the results of the study showed that the mean weight loss in the intervention group was significant. The mean daily calorie intake, fat, and carbohydrate consumption were significantly reduced. The frequency of brisk walking exercise was significantly increased. The frequency of exercise at home increased. Finally, the researchers suggested that this intervention could be integrated into primary care and fertility medical visits as an exclusive treatment or in combination with infertility treatment for infertile women with PCOS.^(39)^ Although the nutritional status of the participants was not examined in the present study, improvements in the lifestyle affecting weight can relatively confirm the improvement in the studied individuals’ food intake.

However, it should be noted that the effectiveness of MI intervention may be affected by cultural differences, social structures, and health care systems in different countries. Studies have shown that MI has high cultural adaptability due to its participatory nature and focus on individual values and goals; however, different results may be achieved in societies where individual freedom is more limited or health decisions are more influenced by family or collective structures. In countries with a healthcare system based on pharmacological treatments and less focused on behavioral therapy, MI may be less considered as a complementary or even alternative intervention. Therefore, adapting MI to the cultural and structural context of each community, training local professionals, and designing culturally sensitive interventions can increase its applicability and effectiveness.^(40)^

One limitation of the present study is that only the BMI index was used to assess the weight status of the women studied. Using more indicators of body composition assessment, such as fat and muscle percentage, in future studies can be advantageous in assessing weight status and anthropometric indicators in these women. Since the women in the experimental group were aware of the intervention type, the impossibility of blinding was another limitation of this study. Due to time and resource limitations in this study, the effect of the intervention was measured for up to 12 weeks; however, measuring the long-term effects of MI on lifestyle, weight, and BMI in women with PCOS in future studies can help to better understand the sustainability of the results of this intervention.

## Conclusion

The MI approach has a positive effect on improving weight-related lifestyle in women with PCOS and reducing weight and BMI in these women. PCOS is the most common endocrine and metabolic disorder in women of reproductive age, which, given the burden of this disease, requires global attention to control. MI is a counseling approach to change behavior and increase self-efficacy in overweight women with PCOS, which can improve weight-related lifestyle and weight conditions in these women. Therefore, it is suggested that the MI approach be used in health and treatment centers to strengthen the internal motivation of individuals to promote a weight-related lifestyle and, as a result, weight management and help to better control this disease.

## Data Availability

The research data are described in the article presented.
